# DDX3X and Stress Granules: Emerging Players in Cancer and Drug Resistance

**DOI:** 10.3390/cancers16061131

**Published:** 2024-03-12

**Authors:** Han Zhang, Paula M. Mañán-Mejías, Hannah N. Miles, Andrea A. Putnam, Leonard R. MacGillivray, William A. Ricke

**Affiliations:** 1 Division of Pharmaceutical Sciences, School of Pharmacy, University of Wisconsin-Madison, Madison, WI 53705, USA; 2 Department of Biomolecular Chemistry, School of Medicine and Public Health, University of Wisconsin-Madison, Madison, WI 53705, USA; 3 Department of Chemistry, University of Iowa, Iowa City, IA 52242, USA; 4 Department of Urology, School of Medicine and Public Health, University of Wisconsin-Madison, Madison, WI 53705, USA; 5 George M. O’Brien Urology Research Center of Excellence, School of Medicine and Public Health, University of Wisconsin-Madison, Madison, WI 53705, USA

**Keywords:** DDX3X, stress granule, drug resistance, castration resistance, novel therapy, translational repression, double-negative prostate cancer

## Abstract

**Simple Summary:**

The prevalence of drug resistance has become a global concern, impacting the effectiveness of various cancer treatments. One of the mechanisms of drug resistance involves cellular stress response, in which stress granules (SGs) form against pharmacological stress. Recent research pointed out that an SGs component, DEAD-box helicase 3 X-linked (DDX3X) protein, is upregulated in multiple cancers, but its role in cancer drug resistance has yet to be fully understood. This review aims to describe the relationship among DDX3X, SGs, and drug resistance in depth.

**Abstract:**

The DEAD (Asp-Glu-Ala-Asp)-box helicase 3 X-linked (DDX3X) protein participates in many aspects of mRNA metabolism and stress granule (SG) formation. DDX3X has also been associated with signal transduction and cell cycle regulation that are important in maintaining cellular homeostasis. Malfunctions of DDX3X have been implicated in multiple cancers, including brain cancer, leukemia, prostate cancer, and head and neck cancer. Recently, literature has reported SG-associated cancer drug resistance, which correlates with a negative disease prognosis. Based on the connections between DDX3X, SG formation, and cancer pathology, targeting DDX3X may be a promising direction for cancer therapeutics development. In this review, we describe the biological functions of DDX3X in terms of mRNA metabolism, signal transduction, and cell cycle regulation. Furthermore, we summarize the contributions of DDX3X in SG formation and cellular stress adaptation. Finally, we discuss the relationships of DDX3X, SG, and cancer drug resistance, and discuss the current research progress of several DDX3X inhibitors for cancer treatment.

## 1. Introduction

**Figure 1:**
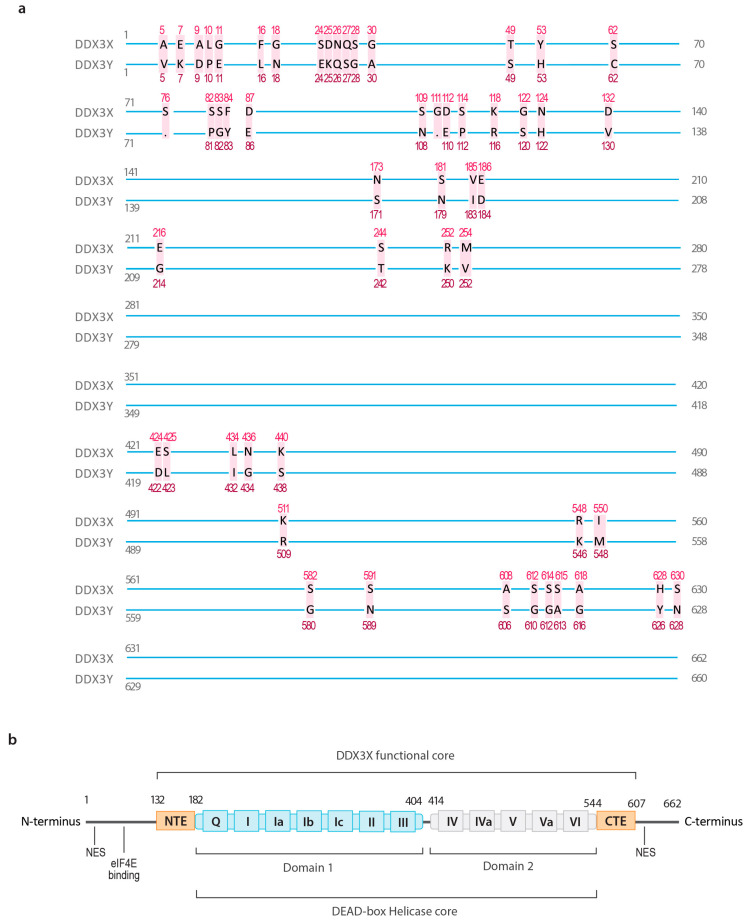
Schematics for DDX3X and DDX3Y. (**a**) Human isoform 1 DDX3X and DDX3Y proteins share ~92% identity in their amino acid sequences. The amino acids that are different between DDX3X and DDX3Y are indicated in red. (**b**) DDX3X contains two RecA-like domains that construct the conservative Asp-Glu-Ala-Asp (DEAD)-box helicase core. Within the domains, there are twelve signature motifs that are responsible for ATP binding and hydrolysis (motifs Q, I, II, and VI), RNA binding (motifs Ia, Ib, Ic, IV, IVa, and V), and RNA/ATP recognition (motifs III and Va). Two nuclear export signals (NESs) have been identified at both the N- and C- terminus, with an eIF4E-binding site located at the N-terminus. NTE, N-terminal extension; CTE, C-terminal extension.

RNA helicases are enzymes that regulate nearly all aspects of RNA metabolism, including transcription, splicing, export, and translation, in an ATP-dependent manner [1,2]. The largest family of RNA helicases are the DEAD-box proteins, which feature a highly conserved Asp-Glu-Ala-Asp (DEAD) amino acid sequence [3]. Within this family, DEAD-box helicase 3 (DDX3) is widely expressed in eukaryotes, and malfunctions of human DDX3 are implicated in multiple diseases [3,4]. There are two paralogs of *DDX3* genes in the human genome: *DDX3X* and *DDX3Y*. While *DDX3Y* is encoded on the Y-chromosomal Azoospermia Factor a (AZFa) region (q11.21) and only expressed in males, *DDX3X* is located on the X chromosome p11.4 expressed in both males and females, and is known to escape X inactivation in females [5,6,7]. DDX3X and DDX3Y proteins share ~92% identity in their amino acid sequences; however, their functions are different in numerous tissues (Figure 1a) [6]. DDX3X participates in RNA metabolism, signal transduction, cell cycle regulation, chromatin remodeling, and stress response [3,6,8,9,10,11]. DDX3X mutations are implicated in the pathology of cancers (e.g., brain cancer, leukemia, and head and neck cancer) and neurodevelopmental disorders (e.g., intellectual disability and autism) [3,4]. Historically, it was believed that DDX3Y only exists in male reproductive tissues; however, a recent study has shown that DDX3Y is expressed in 25 human tissues [5,12]. Furthermore, the deletion of DDX3Y can lead to infertility in males and impaired neural development, suggesting that it is expressed and plays important roles in other tissues [13,14].

DDX3X is a conserved component of stress granules (SGs) and modulates SG assembly, a type of non-membrane-bound messenger ribonucleoprotein (mRNP) granule that forms under stressful conditions in the cytoplasm [15]. The formation of SGs is important to maintain cellular homeostasis during stress, and multiple effects of SG have been identified, including translation repression and stress signaling [16,17]. Further cancer-associated mutations in DDX3X can drive SG assembly [18]. In this review, we first describe the structure and biological functions of DDX3X. Then, we discuss the function of SGs in stress adaptation and the role of DDX3X in SG formation. Finally, we discuss the development of DDX3X-mediated drug resistance in the context of SG. Our analysis will provide a more comprehensive understanding of the roles of DDX3X and SGs in tumorigenesis, and we will conclude with thoughts about future therapeutic targets.

## 2. Overview of DDX3X

### 2.1. The Structure of DDX3X

The structure of DDX3X is highly conserved among different species, suggesting that DDX3X plays an important role in biological functions [6]. The full-length DDX3X protein is composed of 662 amino acids, with a molecular weight of ~73 kDa [19]. As a member of the DEAD-box helicase family, DDX3X contains 12 motifs that form two highly conserved RecA-like domains (domain 1 and domain 2) [6]. These two domains are spaced by 10 amino acids, and perform the functions of ATP binding and hydrolysis (motifs Q, I, II, and VI), RNA binding (motifs Ia, Ib, Ic, IV, IVa, and V), and communication with other molecules (motifs III and Va) (Figure 1b) [3,6]. DDX3X is a nucleic acid-binding protein and binds to double-stranded RNA (dsRNA), single-stranded RNA (ssRNA), double-stranded DNA (dsDNA), and single-stranded DNA (ssDNA) [20,21].

Different from most DEAD-box helicases, the helicase core (domain 1 and domain 2) without flanking N- or C-terminal sequences does not have ATPase activity in vitro [19,22]. Additional studies have shown the N-terminal extension (NTE; residues 132–168) and the C-terminal extension (CTE; residues 582–607) are essential to DDX3X helicase activity, which redefined the minimal functional core of DDX3X [23]. One crystallography study found that the RecA domains of DDX3X with the NTE and CTE dimerize during the recognition of dsRNA, with each DDX3X recognizing a single RNA strand [24]. Subsequent ATP binding to DDX3X induces dsRNA unwinding [24]. The NTE and CTE have been proposed to enhance ATP and RNA binding to DDX3X, respectively [24]. Specifically, it has been revealed that an N-terminal ATP-binding loop (residues 152–160) is required for RNA-stimulated DDX3X-ATP interaction, while the removal of the RDYR motif from the CTE weakens RNA unwinding [23]. Nuclear export signals have been identified in the C- and N-terminal domains, and DDX3X can be both cytoplasmic and nuclear depending on the tissue type [25]. Additionally, the N-terminal domain contains an eIF4E-binding motif [26]. Together, these findings suggest that future studies on the flanking regions could be helpful in further interpreting the molecular role of DDX3X.

### 2.2. The Biological Functions of DDX3X in Normal Physiology and Diseases

#### 2.2.1. mRNA Transcription

**Figure 2:**
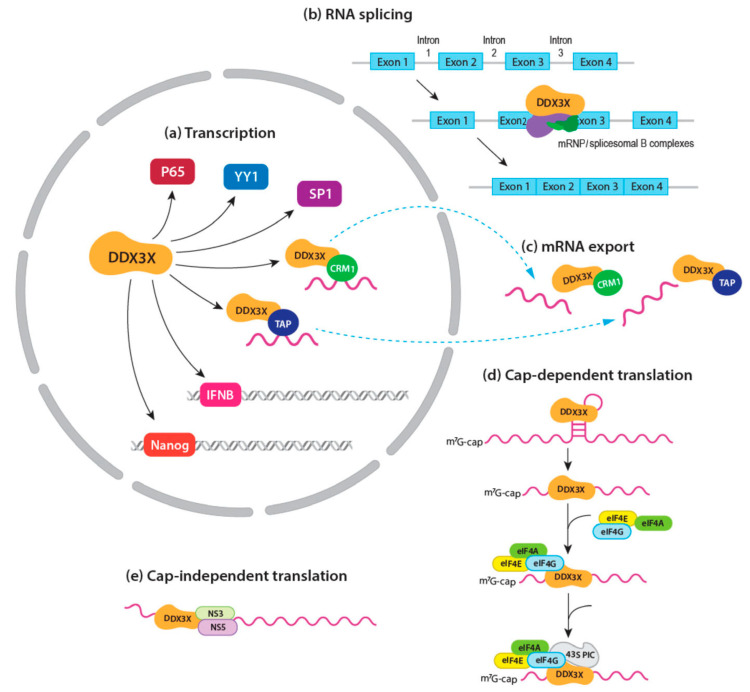
Roles of DDX3X in mRNA metabolism. (**a**) DDX3X regulates transcription through its interactions with transcription factors such as p65, Yin Yang 1 (YY1), and SP1. DDX3X can also directly bind to *IFNB* and *Nanog* promoters to manipulate transcription. (**b**) DDX3X has been identified in human messenger ribonucleoproteins (mRNPs) and spliceosomal B complexes in physiological environments, which are involved in mRNA splicing. The green and purple shapes indicate DDX3-binding molecules in mRNPs and spliceosomal B. (**c**) DDX3X promotes mRNA export via binding to mRNA-exporting proteins CRM1 and Tip-associated protein (TAP). (**d**) DDX3X is able to unwind the secondary structure of mRNA, and promotes ribosome access to mRNA during cap-dependent translation. Currently, it is widely accepted that the translational regulation of DDX3X is mediated by eukaryotic translation initiation factors (eIFs). (**e**) Cap-independent translation usually occurs under stress conditions or during viral infection. In Japanese encephalitis virus (JEV) infection, DDX3X is bound to JEV non-structural protein 3 (NS3), NS5, as well as viral RNA.

DDX3X participates in almost every step of mRNA synthesis and processing. One of the best characterized functions of DDX3X-mediated transcriptional regulation is through its interaction with transcription factors (Figure 2a). For example, DDX3X binds to the transcription factor SP1 and increases the binding affinity of SP1 to the murine double minute 2 (*MDM2*) promoter [27,28]. Other SP1-targeted promoters under DDX3X regulation include the *p21* promoter and *KRAS* promoter [29,30]. Moreover, DDX3X is shown to regulate transcription factors Yin Yang 1 (YY1) and p65 at their zinc finger (ZnF) domain and Rel homology (RH) domain, respectively [31,32]. DDX3X can also directly interact with promoters, such as *IFNB* and *Nanog* promoters, without involving transcription factors [33,34]. However, a recent study in non-plasmacytoid dendritic (pDC) cells indicated that the *IFNB* promoter can be regulated by DDX3X via the IRF-3/p300 holocomplex as well [35]. The direct binding of DDX3X to promoters further supports that DDX3X is able to recognize dsDNA.

#### 2.2.2. mRNA Splicing

There is a scarcity of published studies on the role of DDX3X in mRNA splicing (Figure 2b). Although extensive research has been carried out involving DDX3X knockdown/knockout, no single study has reported defects in pre-mRNA splicing as a result. Nevertheless, analysis using mass spectrometry (MS) identified DDX3X as a protein component of human messenger ribonucleoproteins (mRNPs) and spliceosomal B complexes in physiological environments [36,37]. Further research indicated, however, that DDX3X is co-localized with exon junction complex (EJC) proteins only within spliced mRNPs [36]. Whether DDX3X plays a role in splicing has yet to be determined.

#### 2.2.3. mRNA Export

DDX3X facilitates mRNA nucleo-cytoplasmic shuttling along with other proteins in mRNPs (Figure 2c). For instance, DDX3X directly interacts with the human immunodeficiency virus (HIV) protein Rev and cellular protein CRM1 to escort viral transcripts through the nuclear membrane [38,39]. The N-terminal NES of DDX3X is required for DDX3X-CRM1 binding, which exports DDX3X to the cytoplasm [40,41]. Knockdown of DDX3X suppresses HIV-1 replication by inhibiting mRNA nuclear exporting [42]. Another CRM1-mediated mRNA export mechanism is specific to a subset of cyclin mRNAs that have a translation initiation factor 4E (eIF4E)-sensitive element at their 3′ untranslated regions (UTRs) [43]. DDX3X binds to eIF4E at the N-terminal eIF4E binding site, resulting in an RNase-resistant export mRNP [43]. In addition, DDX3X interacts with Tip-associated protein (TAP), a well-known mRNA export protein [44]. TAP mediates mRNA export by docking to the nuclear pore complex (NPC) via its C-terminal domains [44]. This process is initiated from the recruitment of TAP to EJC-containing mRNPs and includes DDX3X as cargo [44]. As noted, DDX3X may first participate in mRNA splicing in the nucleus, then travel to the cytoplasm within mRNPs and fulfill its function in mRNA translation [6]. Additionally, DDX3X is involved in the nuclear translocation of another DEAD-box helicase DDX5. It has been shown that DDX3X directly binds to DDX5 during the cell cycle [45]. The knockdown of DDX3X inhibits the shuttling of DDX5 from the cytoplasm to the nucleus in G2/M phase [45].

#### 2.2.4. mRNA Translation

A well-studied function of DDX3X is its role in translation, and DDX3X-mediated translation initiation has become a hot topic in molecular biology. In eukaryotic cells, DDX3X has been shown to participate in both cap-dependent and cap-independent translation mechanisms. Canonical translation is cap-dependent translation, which requires the presence of a 7-methylguanosine (m7G) cap on the 5′ end of mRNA. Briefly, this process starts with the formation of 43S pre-initiation complexes (PIC), which contain multiple eIFs, including eIF2, eIF3, and eIF5 [46]. The 43S PIC is recruited to mRNA by the eIF4F complex. The eIF4F complex consists of eIF4A, eIF4E, and eIF4G, and docks at the m7G cap of mRNA through interactions of eIF4E with the cap [46]. Next, the 43S PIC scans mRNA 5′UTRs in a 5′ to 3′ direction until the start codon is recognized, resulting in formation of the 48S initiation complex [46]. Finally, the 60S ribosomal subunit joins the 48S complex to assemble an 80S initiation complex, an assembled ribosome, which then initiates the elongation of peptides [46]. DDX3X, as well as its yeast ortholog Ded1, are implicated in cap-dependent translation initiation (Figure 2d). The first systematic study on Ded1 was conducted in 1997, showing that loss-of-function Ded1 leads to translation suppression in yeast [47]. A following study indicated that Ded1 acts as a potent unwinding factor, and facilitates ribosomal scanning at mRNA 5′UTR [48]. Like Ded1, human DDX3X is able to unwind the secondary structure of mRNA, and promotes the access of ribosomes to mRNA. Currently, it is widely accepted that translational regulation by DDX3X is mediated through interactions with eIFs. It has been demonstrated that DDX3X interacts with eIF3, a component of the 43S PIC [49]. Genetic inhibition of DDX3X resulted in decreased cell viability and impeded mRNA translation in both human HeLa cells and Drosophila [49]. Another study was published that supported the importance of DDX3X in cap-dependent translation with human hepatocellular carcinoma Huh7 cells. DDX3X was observed to interact with eIFs and the 40S ribosomal subunit, facilitating the assembly of the 80S ribosomal complex [9]. Moreover, a study in 2012 indicated that DDX3X directly interacts with eIF4G in the eIF4F complex [50]. However, other members of the eIF4F complex, such as eIF4A or eIF4E, do not interact with DDX3X in the context of translation initiation [50]. In addition, several studies showed that the knockdown of DDX3X did not affect general mRNA translation, but did affect the translation of a subset of mRNAs, suggesting that DDX3X regulates translation with co-factor and mRNA structure specificities [3,50].

In contrast to the cap-dependent translation, the cap-independent translation does not require an intact cap structure at the 5′UTR of the mRNA molecule. Instead, the internal ribosome entry site (IRES) is present in the 5′UTR. This type of translation usually occurs under stress conditions or during viral infection, even though the canonical cap-dependent translation is not completely unavailable [51,52]. DDX3X has been shown to be a pro-viral factor in many cases of viral infection by promoting cap-independent translation (Figure 2e). For example, during Japanese encephalitis virus (JEV) infection, DDX3X is bound to JEV non-structural protein 3 (NS3), NS5, as well as viral RNA [53]. Genetic knockdown of DDX3X using shRNA resulted in a significant reduction in the JEV genomic RNA level, indicating that JEV replication in host cells relies on the helicase activity of DDX3X [53]. Other viruses targeting DDX3X during infection include but are not limited to HIV, hepatitis B virus (HBV), hepatitis C virus (HCV), and SARS-CoV-2 [10,54]. 

To summarize, DDX3X is widely involved in mRNA metabolism and plays an important role in transcription, mRNA splicing, mRNA export, and translation. DDX3X-mediated regulation depends on not only the helicase core but also the N- and C-terminal domains. However, because of its broad range of functions, the molecular mechanisms of DDX3X in normal physiology and disease are not fully understood and need further elucidation.

#### 2.2.5. Signal Transduction

**Figure 3:**
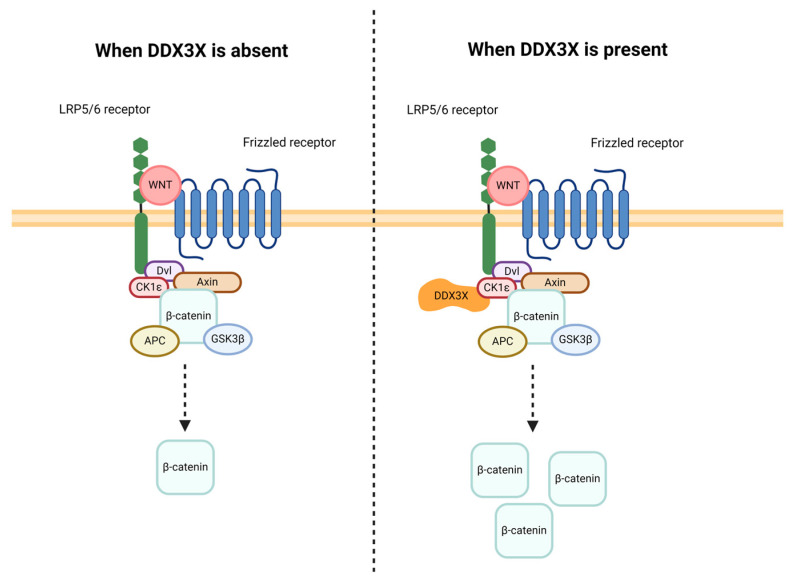
DDX3X enhances Wnt signaling. DDX3X enhances CK1ε activity by direct binding at the C-terminal subdomain, leading to an accumulation of β-catenin. CK1ε, casein kinase 1; Dvl, disheveled; APC, adenomatous polyposis coli; GSK3β, glycogen synthase kinase-3β.

DDX3X is implicated in multiple signaling pathways, of which the Wnt/β-catenin signal transduction cascade is key. Wnt signaling is an indispensable regulator in cellular homeostasis, proliferation, differentiation, cell migration, and apoptosis [55,56]. Malfunction of Wnt signaling is associated with multiple diseases, including cancers, neurodegenerative diseases, and metabolic disorders [56]. In short, when the Wnt/β-catenin signaling is not activated, the destruction complex that consists of casein kinase 1 (CK1ε), Axin, dishevelled (Dvl), adenomatous polyposis coli (APC), and glycogen synthase kinase-3β (GSK3β) sequesters β-catenin in the cytoplasm, phosphorylating β-catenin for degradation [57]. After Wnt binds to the Frizzled (Fz)-lipoprotein receptor-related protein 5/6 (LRP5/6) receptor complex, the destruction complex of β-catenin is inactivated by inhibiting GSK3β phosphorylation, leading to an accumulation of intracellular β-catenin [56]. The elevated level of β-catenin further causes the engagement of transcription factors in the nucleus [56]. One recent study showed that DDX3X modulates CK1ε by direct binding at the C-terminal subdomain in a Wnt-dependent manner (Figure 3) [58]. Genetic inhibition of DDX3X reduces CK1ε activity and affects Wnt/β-catenin signaling in both human cells and *Xenopus* [58]. A follow-up study indicated that RNA binding to DDX3X results in a decreased CK1ε-DDX3X binding affinity [59]. Moreover, carcinogenic mutations of the *DDX3X* gene lead to enhanced CK1ε activity [59]. These findings suggest that DDX3X is positively correlated with CK1ε activity in Wnt/β-catenin signaling, which could be a potential drug target in cancer therapeutics. DDX3X can also regulate Wnt/β-catenin signaling via Rac Family Small GTPase 1 (Rac1). A paper published in 2015 revealed that DDX3X upregulates the translation of Rac1 dependent on helicase activity, which then sustains β-catenin activity by preventing its degradation [60]. This regulation requires an intact 5′ UTR in Rac1 mRNA [60]. Also, the researchers showed that the overexpression of Rac1 is able to rescue β-catenin deficiency in DDX3X-depleted cells [60]. In addition, even though DDX3X does not regulate β-catenin translation, it facilitates the expression of β-catenin via the physical interaction with transcription factors. For instance, DDX3X-bound transcription factor YY1 undergoes transactivation and initiates the transcription of genes associated with β-catenin activation [31]. 

Another process involving DDX3X is the epithelial–mesenchymal transition (EMT). During EMT, the cell morphology and protein expression change dramatically. While the epithelial cells are non-migratory and usually connected via adherens and tight junctions, the mesenchymal cells can migrate along the extracellular matrix [61]. Moreover, during the transition, the expressions of epithelial markers such as E-cadherin, claudins, occludins, and cytokeratins decrease, whereas the expressions of mesenchymal markers such as N-cadherin, vimentin, and fibronectin increase [61]. These changes make cells more migratory and are important to embryonic development, tissue remodeling, and damage repair under normal conditions [57]. However, the loss of adhesion also allows cancer cells to leave the site of the primary tumor, enhancing cancer invasion and metastasis. Thus, targeting the EMT process is a promising mechanism to reduce metastatic progression. A study in 2011 indicated that the overexpression of DDX3X repressed E-cadherin expression in a hypoxia-inducible factor 1 (HIF1)-dependent manner in breast cancer [62]. Similarly, DDX3X reduces E-cadherin expression by upregulating the expression of an E-cadherin repressor Snail in breast cancer cell line MCF7 and several colorectal cancer cell lines [63,64]. Additionally, DDX3X is able to, in turn, regulate HIF1 indirectly through KRAS. As the expression of DDX3X is induced by HIF1, the overexpressed DDX3X then enhances the binding of transcription factor SP1 to the *KRAS* promoter, generating a KRAS/HIF-1α/DDX3 axis feedback loop [65]. However, another study using breast cancer patient samples found a positive correlation between DDX3X and E-cadherin without the involvement of HIF1 [66]. These results suggest the functions of DDX3X in EMT are complex and it may act differently with/without the presence of HIF1, although further validation is needed. 

#### 2.2.6. Cell Cycle Regulation

DDX3X plays a part in cell cycle regulation during normal embryogenesis and abnormal tumorigenesis by manipulating the expressions of cyclins, cyclin-dependent kinases (CDKs), and cyclin-dependent kinases inhibitors (CKIs) [6]. DDX3X can act as an enhancer of proliferation, and upregulated DDX3X expression has been observed in multiple cancers [3]. In breast cancer, DDX3X has been found to inhibit the expression of the transcription factor Kruppel-like factor 4 (KLF4) [67]. Knockdown of DDX3X rescued KLF4 expression and resulted in reduced expressions of cell division-related genes [67]. Such genes include cyclin A2 (*CCNA2*) and *CDK2* [67]. Other groups also reported that the genetic and pharmacological knockdown of DDX3X caused delayed cell cycle progression in different cell lines and organisms. The mechanisms behind this include impaired G1/S phase transition due to inhibited expressions of cyclin A1, cyclin D1, cyclin E1, and CDK2 [68]. Compared with other mRNAs, such cyclin-encoding mRNAs often have complex 5′UTR structures, making them more sensitive to DDX3X deficiency [8,68]. Contrarily, DDX3X can also inhibit cell proliferation through transcriptional regulation. For example, DDX3X reduces tumorigenesis by increasing transcription factor Sp1 binding affinity to p21 the promoter, which further induces cell cycle arrest by inactivating CDK complexes [30]. In a word, DDX3X is a multifaceted effector in cell cycle regulation. A better understanding of its regulatory mechanisms could facilitate the development of novel cancer therapeutics.

#### 2.2.7. Stress Response

Stress from physiological and pathological changes is common, and often affects cellular homeostasis in eukaryotic cells. Such stress includes hypoxic stress, metabolic stress, and therapeutic stress [11]. One pivotal aspect of stress responses is translational adaptation, where SGs form in response to the release of mRNAs from ribosomes upon inhibition of translation. SGs are membrane-less cytoplasmic structures consisting of proteins and mRNAs, also known as mRNPs [69]. Currently, a number of studies have provided detailed information about the composition of SGs. First, mRNAs in SGs are usually untranslated or poorly translated [70,71]. Second, only the 40S, not the 60S, ribosomal subunit is observed in SGs, which suggests the incomplete assembly of the 80S ribosomal translation initiation complex may mediate mRNA entry into SGs [72]. 

While several studies, have shown that DDX3X is important for SG formation and dynamics, the DDX3X-mediated mechanisms by which SGs are regulated have not been well established. Currently, several SG proteins such as eIF4E and polyadenylate-binding protein 1 (PABP1) have been identified to interact with DDX3X by immunoprecipitation, while many others are shown to co-localize with DDX3X at SGs [26,73]. Additionally, the role of DDX3X may be contingent on specific conditions. For example, the knockdown of DDX3X did not yield a significant impact on SG assembly, whereas pharmacological inhibition of DDX3X restricted SG formation in human osteosarcoma U2OS cells [74,75]. Another study suggests that DDX3X can promote the formation of SGs through interactions with other RNA-binding proteins (RBPs) [26]. DDX3X silencing or impeded DDX3X-eIF4E interaction attenuated the formation of SGs [26]. Furthermore, recent literature revealed that the pharmacological inhibition of DDX3X by small molecules RK-33 and 16D significantly reduced SG assembly [74]. Interestingly, the disassembly of SGs is marginally influenced by DDX3X repression through small molecule inhibitors [74]. Based on these observations, DDX3X may play a diverse role in facilitating SG formation under different conditions, depending on the specific RBPs involved. Further research is needed to fully understand the nuanced role of DDX3X in stress granule formation. 

In addition to their role in SGs, DDX3X is involved in other stress responses occurring in inflammation and DNA damage. A comprehensive study indicated that DDX3X modulates the expression of a set of inflammatory genes, including transforming growth factor-β-activated kinase 1 (*TAK1*), interleukin-15 (*IL15*), C-C motif chemokine ligand 5 (*CCL5*), and interferon beta (*IFNβ*) [76]. In bone marrow-derived macrophages, it has been shown that DDX3X interacts with NOD-like receptor family pyrin domain containing 3 (NLRP3) at the NLRP3 NACHT domain, and drives the formation of NLRP3 inflammasomes under stressful conditions [77]. Loss of DDX3X attenuates the assembly of NLRP3 inflammasomes in vitro and in vivo [77]. Since the activation of inflammasomes can lead to programmed cell death, DDX3X is pivotal in the determination of cell fate. DDX3X is also implicated in the DNA damage response, and previous studies demonstrated that DDX3X dysfunction results in accumulated DNA breaks [78]. A follow-up study revealed that DDX3X co-localizes with proteins that are associated with DNA repair in the nucleus [79]. It has also been reported that DDX3X regulates the expression of DNA repair genes [79]. The biogenesis of transfer RNA-derived small RNA (tsRNA) is a cellular stress response that can be induced by DNA damage, hypoxia, and nutrition deprivation [80]. One of the main nucleases involved in this process is angiogenin (ANG). Together with other DEAD-box proteins such as DDX1 and DDX5, DDX3X has been found to facilitate unwinding ANG-processed tRNAs [73]. If cells fail to rescue stress-induced damage, they are destined to programmed cell death. Multiple papers have reported that mutations of DDX3X promote tumorigenesis due to imbalanced proliferation and apoptosis [3,27,81].

## 3. DDX3X and Drug Resistance in Cancer

### 3.1. Overview of Drug Resistance Mechanisms in Cancer

Regardless of tissue type, cancerous cells mutate to bypass cellular growth checkpoints, thus allowing for continuous growth, proliferation, and metabolism [82]. In turn, many broad-spectrum chemotherapeutics target cell division, metabolism, or induce DNA damage that leads to cellular apoptosis. While these chemotherapies have efficacy in certain patient populations, many develop further resistance to these treatments and require additional interventions, with certain subsets developing multiple resistance mechanisms [83]. These modes of resistance include tumor heterogeneity, genetic alterations, drug inactivation, drug efflux, inhibition of apoptosis, and alterations in DNA repair [84]. Some cancer cells remain persistent even without the development of such resistance mechanisms, yet remain long enough to withstand treatment strategies, and thus allow cancer recurrence. Unfortunately, little is known surrounding persistent cells and what biological mechanisms allow for their treatment evasion. Because of this, we will focus our discussion on the putative roles that DDX3X plays in drug resistance across cancer types.

### 3.2. The Role of DDX3X in Drug Resistance

Due to its versatile functionality in cellular biology, DDX3X has been investigated in a variety of cancer types such as melanoma, breast, and prostate cancers; however, its role across cancer types is conflicting [11,85,86,87]. *DDX3X* was first discovered as an oncogene in a genetic screening of cellular transforming genes in hepatocarcinogenesis [88]. The role of DDX3X was investigated in a breast cancer study, where it was found to have an oncogenic role in breast cancer biogenesis [89]. In that study, MCF 10A cells were exposed to the carcinogen Benzo[a]pyrene diol, and gene expression effects were measured. Overexpressed DDX3X resulted, and in turn led to increases in motility and EMT in these breast cell lines [89]. A separate study in lung cancer cells harboring epidermal growth factor receptor (EGFR)-activating mutations found that preferential expression of DDX3X induced a cancer stem cell-like phenotype, increasing EMT as well as a loss of sensitivity to EGFR-tyrosine kinase inhibitors [90]. Overexpression of DDX3X in these lung cells prevented phosphorylation of EGFR-Tyr residues, and instead mediated Wnt/β-catenin signaling; however, the authors were unable to discern exactly how DDX3X inhibits this phosphorylation. This involvement in the β-catenin pathway was further mediated in a separate study examining multiple cancer lines and the effect of DDX3X depletion, as siRNA knockdown of DDX3X reduced cell motility and metastatic potential [60]. Additionally, knockdown reduced levels of β-catenin and Rac1 proteins as well as downstream target genes, confirming the interaction of DDX3X with the Rac1/β-catenin pathway in cancer progression and indicating a potential therapeutic target for tumors with increased Wnt activity.

DDX3X has also been shown to play a role in post-transcriptional mediation of drug resistance. An unbiased translational screening of melanoma phenotypes uncovered the microphthalmia-associated transcription factor (MITF) as a key downstream target of DDX3X in melanomas [91]. By promoting mRNA translation, DDX3X can increase MITF protein levels and alter metastatic potential and response to targeted melanoma therapies. 

Taken together, these studies indicate that high expression of DDX3X in certain cancer types may indicate more aggressive disease, meriting further investigation into DDX3X as both a biomarker of disease severity and an emerging therapeutic target.

### 3.3. DDX3X-Mediated Regulation of Stress Granules in Drug Resistance

**Figure 4:**
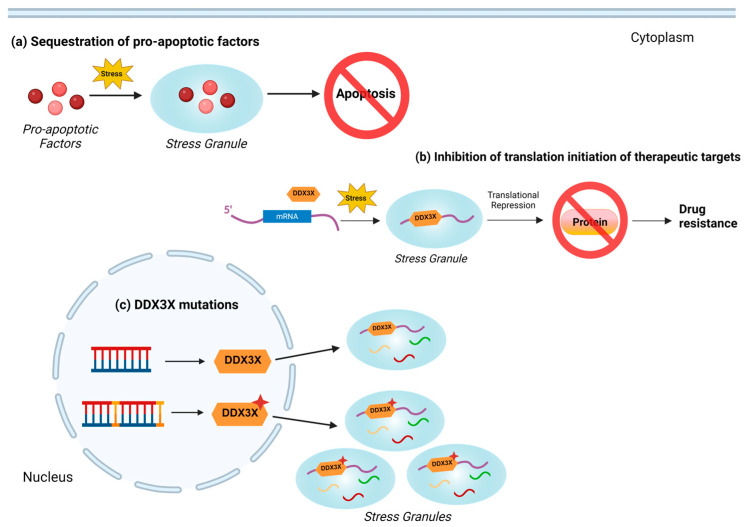
Mechanisms of DDX3X-mediated regulation of stress granules in drug resistance. (**a**) Under stress, stress granules (SGs) can sequester pro-apoptotic factors and prevent apoptosis. (**b**) DDX3X can bind to target mRNA and capture it in SGs, resulting in translational repression. Then, the absence of therapeutic targets leads to drug resistance. (**c**) In medulloblastoma, DDX3X mutations lead to increased SGs assembly, enhancing the mechanisms described in (**a**,**b**).

SGs can lead to drug resistance by limiting cell death; however, the mechanisms are not fully understood. One of the described mechanisms is that SGs help cancer cells survive under stressful conditions induced by chemotherapeutic drugs. SGs have been suggested to prevent apoptosis by sequestering pro-apoptotic factors [91]. One of the known pathways that is under the regulation of SGs is the receptor of the activated protein C kinase 1 (RACK1)/p38/c-Jun N-terminal kinase (JNK) pathway (Figure 4a). During stress, the pleiotropic adaptor protein RACK1 is sequestered in SGs, resulting in inhibited multimerization of the protein kinase MAP 3 kinase 1 (MTK1) [92]. Since MTK1 is an upstream activator of apoptosis inducers p38 and JNK, the sequestration of RACK1 can protect stressed cells from programmed cell death [15]. Likewise, SGs trap mammalian targets of rapamycin complex 1 (mTORC1) under stressful conditions, thereby repressing mTORC1-induced apoptosis [93]. It has been shown that suppressed SGs formation promotes apoptosis and makes cells more sensitive to stress [15]. Cells escape apoptosis which promotes resistance to the chemotherapeutic drugs [94]. Moreover, it has been demonstrated that DDX3X binds to mRNA-encoding therapeutic targets and sequesters it in SGs (Figure 4b). This has been observed in castration-resistant prostate cancer (CRPC), an advanced prostate cancer that becomes resistant to existing therapies that target AR signaling [11]. The researchers found that overexpressed DDX3X protein bound to and sequestered AR mRNA in SGs, inhibiting translation and AR protein expression [11]. Low levels of AR protein prevented the use of common antiandrogen therapies and led to CRPC, thus highlighting the roles of DDX3X and SGs in evading treatment strategies.

Another mechanism by which DDX3X plays a role in SG formation and drug resistance is through DDX3X mutations (Figure 4c). Dysregulation of protein synthesis by mutated DDX3X has been observed in medulloblastoma (MB) [18]. Researchers determined that DDX3X mutations in MB lead to an increase in SG assembly and inhibit mRNA translation by binding to target mRNAs and blocking translation initiation [18]. Currently, the roles of DDX3X and SGs as well as their relationship are still being investigated. Further understanding of the mechanism behind DDX3X-mediated SGs is necessary to identify its role in drug resistance and uncover strategies for anti-cancer therapies. 

### 3.4. Implications of DDX3X Inhibitors in Cancer Treatment

The formation of SGs by DDX3X can be interrupted by pharmacological compounds [74]. DDX3X inhibitors have been shown to be successful in limiting the growth of several cancers and reducing DDX3X-mediated SG assembly. For example, RK-33 has been designed to bind to the ATP-binding domain of DDX3X [95]. It was proposed that the central diazepine ring of RK-33 interacts with the Q motif residue Tyr200 of DDX3X through π–π stacking interactions [96]. It has been effective in inhibiting DDX3X helicase activity in breast, lung, and prostate cancers [95]. RK-33 also inhibits DDX3X during viral infections, presenting as a potential target for antiviral therapy [94]. A second DDX3X inhibitor that has been identified is Ketorolac salt, commercially known as Toradol. It targets DDX3X in oral cancer, inducing cancer cell death and downregulating DDX3X expression [97]. Further efforts should be put towards the development of DDX3X inhibitors or co-treatments as promising candidates for cancer therapies in the future.

## 4. Conclusions

In this review, we have discussed the biological functions of DDX3X, as well as its pathological role in the development of cancer drug resistance. First, DDX3X regulates numerous processes in mRNA metabolism under normal conditions. DDX3X interacts with multiple transcription factors and promoters to regulate mRNA transcription [30,32,33]. Along with other RNA-binding proteins, DDX3X facilitates mRNA export and translation in an ATP-dependent manner [10,43,45,50]. Second, DDX3X mediates multiple cellular signaling transductions, and some of these pathways can promote cancer progression and metastasis [42,60,65,77]. Third, DDX3X is of great importance in the assembly of SGs, which protect cells from stressful environments [26]. Due to the protective nature of DDX3X during cellular stress, drug resistance can develop after cancer treatments. Several anti-DDX3X therapies have shown effectiveness in attenuating SG formation and overcoming drug resistance in certain diseases [11,95,97]. However, the detailed molecular mechanisms of DDX3X and SGs in drug resistance are still under investigation, while some findings are controversial.

Altogether, DDX3X is a rising target for cancer therapeutics. Considering the pivotal roles of DDX3X in maintaining normal cellular functions, the side effects of using anti-DDX3X therapeutics need to be carefully evaluated. Further pharmacological studies and clinical trials will shed light on the application of such therapies in the future.

## Data Availability

The data presented in this study are available in this article.

## References

[1] Donsbach P., Klostermeier D. (2021). Regulation of RNA helicase activity: Principles and examples. Biol. Chem..

[2] Linder P., Jankowsky E. (2011). From unwinding to clamping—The DEAD box RNA helicase family. Nat. Rev. Mol. Cell Biol..

[3] Bol G.M., Xie M., Raman V. (2015). DDX3, a potential target for cancer treatment. Mol. Cancer.

[4] Hoye M.L., Calviello L., Poff A.J., Ejimogu N.E., Newman C.R., Montgomery M.D., Ou J., Floor S.N., Silver D.L. (2022). Aberrant cortical development is driven by impaired cell cycle and translational control in a DDX3X syndrome model. eLife.

[5] Venkataramanan S., Gadek M., Calviello L., Wilkins K., Floor S.N. (2021). DDX3X and DDX3Y are redundant in protein synthesis. RNA.

[6] Mo J., Liang H., Su C., Li P., Chen J., Zhang B. (2021). DDX3X: Structure, physiologic functions and cancer. Mol. Cancer.

[7] Cotton A.M., Price E.M., Jones M.J., Balaton B.P., Kobor M.S., Brown C.J. (2015). Landscape of DNA methylation on the X chromosome reflects CpG density, functional chromatin state and X-chromosome inactivation. Hum. Mol. Genet..

[8] Calviello L., Venkataramanan S., Rogowski K.J., Wyler E., Wilkins K., Tejura M., Thai B., Krol J., Filipowicz W., Landthaler M. (2021). DDX3 depletion represses translation of mRNAs with complex 5’ UTRs. Nucleic Acids Res..

[9] Geissler R., Golbik R.P., Behrens S.E. (2012). The DEAD-box helicase DDX3 supports the assembly of functional 80S ribosomes. Nucleic Acids Res..

[10] Ryan C.S., Schroder M. (2022). The human DEAD-box helicase DDX3X as a regulator of mRNA translation. Front. Cell Dev. Biol..

[11] Vellky J.E., McSweeney S.T., Ricke E.A., Ricke W.A. (2020). RNA-binding protein DDX3 mediates posttranscriptional regulation of androgen receptor: A mechanism of castration resistance. Proc. Natl. Acad. Sci. USA.

[12] Uhlen M., Fagerberg L., Hallstrom B.M., Lindskog C., Oksvold P., Mardinoglu A., Sivertsson A., Kampf C., Sjostedt E., Asplund A. (2015). Proteomics. Tissue-based map of the human proteome. Science.

[13] Matsumura T., Endo T., Isotani A., Ogawa M., Ikawa M. (2019). An azoospermic factor gene, Ddx3y and its paralog, Ddx3x are dispensable in germ cells for male fertility. J. Reprod. Dev..

[14] Vakilian H., Mirzaei M., Sharifi Tabar M., Pooyan P., Habibi Rezaee L., Parker L., Haynes P.A., Gourabi H., Baharvand H., Salekdeh G.H. (2015). DDX3Y, a Male-Specific Region of Y Chromosome Gene, May Modulate Neuronal Differentiation. J. Proteome Res..

[15] Marcelo A., Koppenol R., de Almeida L.P., Matos C.A., Nobrega C. (2021). Stress granules, RNA-binding proteins and polyglutamine diseases: Too much aggregation?. Cell Death Dis..

[16] Kedersha N., Stoecklin G., Ayodele M., Yacono P., Lykke-Andersen J., Fritzler M.J., Scheuner D., Kaufman R.J., Golan D.E., Anderson P. (2005). Stress granules and processing bodies are dynamically linked sites of mRNP remodeling. J. Cell Biol..

[17] Hofmann S., Cherkasova V., Bankhead P., Bukau B., Stoecklin G. (2012). Translation suppression promotes stress granule formation and cell survival in response to cold shock. Mol. Biol. Cell.

[18] Valentin-Vega Y.A., Wang Y.D., Parker M., Patmore D.M., Kanagaraj A., Moore J., Rusch M., Finkelstein D., Ellison D.W., Gilbertson R.J. (2016). Cancer-associated DDX3X mutations drive stress granule assembly and impair global translation. Sci. Rep..

[19] Hogbom M., Collins R., van den Berg S., Jenvert R.M., Karlberg T., Kotenyova T., Flores A., Karlsson Hedestam G.B., Schiavone L.H. (2007). Crystal structure of conserved domains 1 and 2 of the human DEAD-box helicase DDX3X in complex with the mononucleotide AMP. J. Mol. Biol..

[20] Garbelli A., Beermann S., Di Cicco G., Dietrich U., Maga G. (2011). A motif unique to the human DEAD-box protein DDX3 is important for nucleic acid binding, ATP hydrolysis, RNA/DNA unwinding and HIV-1 replication. PLoS ONE.

[21] Putnam A.A., Jankowsky E. (2013). DEAD-box helicases as integrators of RNA, nucleotide and protein binding. Biochim. Biophys. Acta.

[22] Soto-Rifo R., Ohlmann T. (2013). The role of the DEAD-box RNA helicase DDX3 in mRNA metabolism. Wiley Interdiscip. Rev. RNA.

[23] Floor S.N., Condon K.J., Sharma D., Jankowsky E., Doudna J.A. (2016). Autoinhibitory Interdomain Interactions and Subfamily-specific Extensions Redefine the Catalytic Core of the Human DEAD-box Protein DDX3. J. Biol. Chem..

[24] Song H., Ji X. (2019). The mechanism of RNA duplex recognition and unwinding by DEAD-box helicase DDX3X. Nat. Commun..

[25] Vellky J.E., Ricke E.A., Huang W., Ricke W.A. (2019). Expression and Localization of DDX3 in Prostate Cancer Progression and Metastasis. Am. J. Pathol..

[26] Shih J.W., Wang W.T., Tsai T.Y., Kuo C.Y., Li H.K., Wu Lee Y.H. (2012). Critical roles of RNA helicase DDX3 and its interactions with eIF4E/PABP1 in stress granule assembly and stress response. Biochem. J..

[27] Wu D.W., Lee M.C., Wang J., Chen C.Y., Cheng Y.W., Lee H. (2014). DDX3 loss by p53 inactivation promotes tumor malignancy via the MDM2/Slug/E-cadherin pathway and poor patient outcome in non-small-cell lung cancer. Oncogene.

[28] Chao C.H., Chen C.M., Cheng P.L., Shih J.W., Tsou A.P., Lee Y.H. (2006). DDX3, a DEAD box RNA helicase with tumor growth-suppressive property and transcriptional regulation activity of the p21waf1/cip1 promoter, is a candidate tumor suppressor. Cancer Res..

[29] Wu D.W., Lin P.L., Cheng Y.W., Huang C.C., Wang L., Lee H. (2016). DDX3 enhances oncogenic KRAS-induced tumor invasion in colorectal cancer via the beta-catenin/ZEB1 axis. Oncotarget.

[30] Wu D.W., Liu W.S., Wang J., Chen C.Y., Cheng Y.W., Lee H. (2011). Reduced p21(WAF1/CIP1) via alteration of p53-DDX3 pathway is associated with poor relapse-free survival in early-stage human papillomavirus-associated lung cancer. Clin. Cancer Res..

[31] Yang F., Fang E., Mei H., Chen Y., Li H., Li D., Song H., Wang J., Hong M., Xiao W. (2019). Cis-Acting circ-CTNNB1 Promotes beta-Catenin Signaling and Cancer Progression via DDX3-Mediated Transactivation of YY1. Cancer Res..

[32] Xiang N., He M., Ishaq M., Gao Y., Song F., Guo L., Ma L., Sun G., Liu D., Guo D. (2016). The DEAD-Box RNA Helicase DDX3 Interacts with NF-kappaB Subunit p65 and Suppresses p65-Mediated Transcription. PLoS ONE.

[33] Gu L., Fullam A., Brennan R., Schroder M. (2013). Human DEAD box helicase 3 couples IkappaB kinase epsilon to interferon regulatory factor 3 activation. Mol. Cell Biol..

[34] Karmakar S., Rauth S., Nallasamy P., Perumal N., Nimmakayala R.K., Leon F., Gupta R., Barkeer S., Venkata R.C., Raman V. (2020). RNA Polymerase II-Associated Factor 1 Regulates Stem Cell Features of Pancreatic Cancer Cells, Independently of the PAF1 Complex, via Interactions With PHF5A and DDX3. Gastroenterology.

[35] Saikruang W., Ang Yan Ping L., Abe H., Kasumba D.M., Kato H., Fujita T. (2022). The RNA helicase DDX3 promotes IFNB transcription via enhancing IRF-3/p300 holocomplex binding to the IFNB promoter. Sci. Rep..

[36] Merz C., Urlaub H., Will C.L., Luhrmann R. (2007). Protein composition of human mRNPs spliced in vitro and differential requirements for mRNP protein recruitment. RNA.

[37] Deckert J., Hartmuth K., Boehringer D., Behzadnia N., Will C.L., Kastner B., Stark H., Urlaub H., Luhrmann R. (2006). Protein composition and electron microscopy structure of affinity-purified human spliceosomal B complexes isolated under physiological conditions. Mol. Cell Biol..

[38] Mahboobi S.H., Javanpour A.A., Mofrad M.R. (2015). The interaction of RNA helicase DDX3 with HIV-1 Rev-CRM1-RanGTP complex during the HIV replication cycle. PLoS ONE.

[39] Frohlich A., Rojas-Araya B., Pereira-Montecinos C., Dellarossa A., Toro-Ascuy D., Prades-Perez Y., Garcia-de-Gracia F., Garces-Alday A., Rubilar P.S., Valiente-Echeverria F. (2016). DEAD-box RNA helicase DDX3 connects CRM1-dependent nuclear export and translation of the HIV-1 unspliced mRNA through its N-terminal domain. Biochim. Biophys. Acta.

[40] Yedavalli V.S., Neuveut C., Chi Y.H., Kleiman L., Jeang K.T. (2004). Requirement of DDX3 DEAD box RNA helicase for HIV-1 Rev-RRE export function. Cell.

[41] Hernandez-Diaz T., Valiente-Echeverria F., Soto-Rifo R. (2021). RNA Helicase DDX3: A Double-Edged Sword for Viral Replication and Immune Signaling. Microorganisms.

[42] Ishaq M., Hu J., Wu X., Fu Q., Yang Y., Liu Q., Guo D. (2008). Knockdown of cellular RNA helicase DDX3 by short hairpin RNAs suppresses HIV-1 viral replication without inducing apoptosis. Mol. Biotechnol..

[43] Culjkovic B., Topisirovic I., Skrabanek L., Ruiz-Gutierrez M., Borden K.L. (2005). eIF4E promotes nuclear export of cyclin D1 mRNAs via an element in the 3’UTR. J. Cell Biol..

[44] Lai M.C., Lee Y.H., Tarn W.Y. (2008). The DEAD-box RNA helicase DDX3 associates with export messenger ribonucleoproteins as well as tip-associated protein and participates in translational control. Mol. Biol. Cell.

[45] Choi Y.J., Lee S.G. (2012). The DEAD-box RNA helicase DDX3 interacts with DDX5, co-localizes with it in the cytoplasm during the G2/M phase of the cycle, and affects its shuttling during mRNP export. J. Cell Biochem..

[46] Jackson R.J., Hellen C.U., Pestova T.V. (2010). The mechanism of eukaryotic translation initiation and principles of its regulation. Nat. Rev. Mol. Cell Biol..

[47] Chuang R.Y., Weaver P.L., Liu Z., Chang T.H. (1997). Requirement of the DEAD-Box protein ded1p for messenger RNA translation. Science.

[48] Marsden S., Nardelli M., Linder P., McCarthy J.E. (2006). Unwinding single RNA molecules using helicases involved in eukaryotic translation initiation. J. Mol. Biol..

[49] Lee C.S., Dias A.P., Jedrychowski M., Patel A.H., Hsu J.L., Reed R. (2008). Human DDX3 functions in translation and interacts with the translation initiation factor eIF3. Nucleic Acids Res..

[50] Soto-Rifo R., Rubilar P.S., Limousin T., de Breyne S., Decimo D., Ohlmann T. (2012). DEAD-box protein DDX3 associates with eIF4F to promote translation of selected mRNAs. EMBO J..

[51] Yang Y., Wang Z. (2019). IRES-mediated cap-independent translation, a path leading to hidden proteome. J. Mol. Cell Biol..

[52] Shatsky I.N., Terenin I.M., Smirnova V.V., Andreev D.E. (2018). Cap-Independent Translation: What’s in a Name?. Trends Biochem. Sci..

[53] Li C., Ge L.L., Li P.P., Wang Y., Dai J.J., Sun M.X., Huang L., Shen Z.Q., Hu X.C., Ishag H. (2014). Cellular DDX3 regulates Japanese encephalitis virus replication by interacting with viral un-translated regions. Virology.

[54] Sharma D., Putnam A.A., Jankowsky E. (2017). Biochemical Differences and Similarities between the DEAD-Box Helicase Orthologs DDX3X and Ded1p. J. Mol. Biol..

[55] Patel S., Alam A., Pant R., Chattopadhyay S. (2019). Wnt Signaling and Its Significance Within the Tumor Microenvironment: Novel Therapeutic Insights. Front. Immunol..

[56] Ng L.F., Kaur P., Bunnag N., Suresh J., Sung I.C.H., Tan Q.H., Gruber J., Tolwinski N.S. (2019). WNT Signaling in Disease. Cells.

[57] Zhao L., Mao Y., Zhou J., Zhao Y., Cao Y., Chen X. (2016). Multifunctional DDX3: Dual roles in various cancer development and its related signaling pathways. Am. J. Cancer Res..

[58] Cruciat C.M., Dolde C., de Groot R.E., Ohkawara B., Reinhard C., Korswagen H.C., Niehrs C. (2013). RNA helicase DDX3 is a regulatory subunit of casein kinase 1 in Wnt-beta-catenin signaling. Science.

[59] Dolde C., Bischof J., Gruter S., Montada A., Halekotte J., Peifer C., Kalbacher H., Baumann U., Knippschild U., Suter B. (2018). A CK1 FRET biosensor reveals that DDX3X is an essential activator of CK1epsilon. J. Cell Sci..

[60] Chen H.H., Yu H.I., Cho W.C., Tarn W.Y. (2015). DDX3 modulates cell adhesion and motility and cancer cell metastasis via Rac1-mediated signaling pathway. Oncogene.

[61] Gonzalez D.M., Medici D. (2014). Signaling mechanisms of the epithelial-mesenchymal transition. Sci. Signal..

[62] Botlagunta M., Krishnamachary B., Vesuna F., Winnard P.T., Bol G.M., Patel A.H., Raman V. (2011). Expression of DDX3 is directly modulated by hypoxia inducible factor-1 alpha in breast epithelial cells. PLoS ONE.

[63] Sun M., Song L., Zhou T., Gillespie G.Y., Jope R.S. (2011). The role of DDX3 in regulating Snail. Biochim. Biophys. Acta.

[64] Su C.Y., Lin T.C., Lin Y.F., Chen M.H., Lee C.H., Wang H.Y., Lee Y.C., Liu Y.P., Chen C.L., Hsiao M. (2015). DDX3 as a strongest prognosis marker and its downregulation promotes metastasis in colorectal cancer. Oncotarget.

[65] Wu D.W., Lin P.L., Wang L., Huang C.C., Lee H. (2017). The YAP1/SIX2 axis is required for DDX3-mediated tumor aggressiveness and cetuximab resistance in KRAS-wild-type colorectal cancer. Theranostics.

[66] Bol G.M., Raman V., van der Groep P., Vermeulen J.F., Patel A.H., van der Wall E., van Diest P.J. (2013). Expression of the RNA helicase DDX3 and the hypoxia response in breast cancer. PLoS ONE.

[67] Cannizzaro E., Bannister A.J., Han N., Alendar A., Kouzarides T. (2018). DDX3X RNA helicase affects breast cancer cell cycle progression by regulating expression of KLF4. FEBS Lett..

[68] Sergeeva O., Zatsepin T. (2021). RNA Helicases as Shadow Modulators of Cell Cycle Progression. Int. J. Mol. Sci..

[69] Ivanov P., Kedersha N., Anderson P. (2019). Stress Granules and Processing Bodies in Translational Control. Cold Spring Harb. Perspect. Biol..

[70] Wang F., Li J., Fan S., Jin Z., Huang C. (2020). Targeting stress granules: A novel therapeutic strategy for human diseases. Pharmacol. Res..

[71] Khong A., Matheny T., Jain S., Mitchell S.F., Wheeler J.R., Parker R. (2017). The Stress Granule Transcriptome Reveals Principles of mRNA Accumulation in Stress Granules. Mol. Cell.

[72] Fedorovskiy A.G., Burakov A.V., Terenin I.M., Bykov D.A., Lashkevich K.A., Popenko V.I., Makarova N.E., Sorokin I.I., Sukhinina A.P., Prassolov V.S. (2023). A Solitary Stalled 80S Ribosome Prevents mRNA Recruitment to Stress Granules. Biochemistry.

[73] Drino A., Konig L., Capitanchik C., Sanadgol N., Janisiw E., Rappol T., Vilardo E., Schaefer M.R. (2023). Identification of RNA helicases with unwinding activity on angiogenin-processed tRNAs. Nucleic Acids Res..

[74] Cui B.C., Sikirzhytski V., Aksenova M., Lucius M.D., Levon G.H., Mack Z.T., Pollack C., Odhiambo D., Broude E., Lizarraga S.B. (2020). Pharmacological inhibition of DEAD-Box RNA Helicase 3 attenuates stress granule assembly. Biochem. Pharmacol..

[75] Yang P., Mathieu C., Kolaitis R.M., Zhang P., Messing J., Yurtsever U., Yang Z., Wu J., Li Y., Pan Q. (2020). G3BP1 Is a Tunable Switch that Triggers Phase Separation to Assemble Stress Granules. Cell.

[76] Ku Y.C., Lai M.H., Lo C.C., Cheng Y.C., Qiu J.T., Tarn W.Y., Lai M.C. (2019). DDX3 Participates in Translational Control of Inflammation Induced by Infections and Injuries. Mol. Cell Biol..

[77] Samir P., Kesavardhana S., Patmore D.M., Gingras S., Malireddi R.K.S., Karki R., Guy C.S., Briard B., Place D.E., Bhattacharya A. (2019). DDX3X acts as a live-or-die checkpoint in stressed cells by regulating NLRP3 inflammasome. Nature.

[78] Heerma van Voss M.R., Brilliant J.D., Vesuna F., Bol G.M., van der Wall E., van Diest P.J., Raman V. (2017). Combination treatment using DDX3 and PARP inhibitors induces synthetic lethality in BRCA1-proficient breast cancer. Med. Oncol..

[79] Cargill M.J., Morales A., Ravishankar S., Warren E.H. (2021). RNA helicase, DDX3X, is actively recruited to sites of DNA damage in live cells. DNA Repair.

[80] Li G., Manning A.C., Bagi A., Yang X., Gokulnath P., Spanos M., Howard J., Chan P.P., Sweeney T., Kitchen R. (2022). Distinct Stress-Dependent Signatures of Cellular and Extracellular tRNA-Derived Small RNAs. Adv. Sci..

[81] Epling L.B., Grace C.R., Lowe B.R., Partridge J.F., Enemark E.J. (2015). Cancer-associated mutants of RNA helicase DDX3X are defective in RNA-stimulated ATP hydrolysis. J. Mol. Biol..

[82] Pavlova N.N., Zhu J., Thompson C.B. (2022). The hallmarks of cancer metabolism: Still emerging. Cell Metab..

[83] Bukowski K., Kciuk M., Kontek R. (2020). Mechanisms of Multidrug Resistance in Cancer Chemotherapy. Int. J. Mol. Sci..

[84] Haider T., Pandey V., Banjare N., Gupta P.N., Soni V. (2020). Drug resistance in cancer: Mechanisms and tackling strategies. Pharmacol. Rep..

[85] Pardeshi J., McCormack N., Gu L., Ryan C.S., Schröder M. (2022). DDX3X functionally and physically interacts with Estrogen Receptor-alpha. Biochim. Biophys. Acta BBA Gene Regul. Mech..

[86] Pan X., Chen G., Hu W. (2021). lncRNA HLA Complex Group 18 (HCG18) Facilitated Cell Proliferation, Invasion, and Migration of Prostate Cancer Through Modulating miR-370-3p/DDX3X Axis. Reprod. Sci..

[87] Alkallas R., Lajoie M., Moldoveanu D., Hoang K.V., Lefrançois P., Lingrand M., Ahanfeshar-Adams M., Watters K., Spatz A., Zippin J.H. (2020). Multi-omic analysis reveals significantly mutated genes and DDX3X as a sex-specific tumor suppressor in cutaneous melanoma. Nat. Cancer.

[88] Huang J.-S., Chao C.-C., Su T.-L., Yeh S.-H., Chen D.-S., Chen C.-T., Chen P.-J., Jou Y.-S. (2004). Diverse cellular transformation capability of overexpressed genes in human hepatocellular carcinoma. Biochem. Biophys. Res. Commun..

[89] Botlagunta M., Vesuna F., Mironchik Y., Raman A., Lisok A., Winnard P., Mukadam S., Van Diest P., Chen J.H., Farabaugh P. (2008). Oncogenic role of DDX3 in breast cancer biogenesis. Oncogene.

[90] Nozaki K., Kagamu H., Shoji S., Igarashi N., Ohtsubo A., Okajima M., Miura S., Watanabe S., Yoshizawa H., Narita I. (2014). DDX3X Induces Primary EGFR-TKI Resistance Based on Intratumor Heterogeneity in Lung Cancer Cells Harboring EGFR-Activating Mutations. PLoS ONE.

[91] Phung B., Cieśla M., Sanna A., Guzzi N., Beneventi G., Cao Thi Ngoc P., Lauss M., Cabrita R., Cordero E., Bosch A. (2019). The X-Linked DDX3X RNA Helicase Dictates Translation Reprogramming and Metastasis in Melanoma. Cell Rep..

[92] Kedersha N., Ivanov P., Anderson P. (2013). Stress granules and cell signaling: More than just a passing phase?. Trends Biochem. Sci..

[93] Thedieck K., Holzwarth B., Prentzell M.T., Boehlke C., Klasener K., Ruf S., Sonntag A.G., Maerz L., Grellscheid S.N., Kremmer E. (2013). Inhibition of mTORC1 by astrin and stress granules prevents apoptosis in cancer cells. Cell.

[94] Yang S.N.Y., Atkinson S.C., Audsley M.D., Heaton S.M., Jans D.A., Borg N.A. (2020). RK-33 Is a Broad-Spectrum Antiviral Agent That Targets DEAD-Box RNA Helicase DDX3X. Cells.

[95] Xie M., Vesuna F., Tantravedi S., Bol G.M., Heerma van Voss M.R., Nugent K., Malek R., Gabrielson K., van Diest P.J., Tran P.T. (2016). RK-33 Radiosensitizes Prostate Cancer Cells by Blocking the RNA Helicase DDX3. Cancer Res..

[96] Kondaskar A., Kondaskar S., Fishbein J.C., Carter-Cooper B.A., Lapidus R.G., Sadowska M., Edelman M.J., Hosmane R.S. (2013). Structure-based drug design and potent anti-cancer activity of tricyclic 5:7:5-fused diimidazo[4,5-d:4′,5′-f][1,3]diazepines. Bioorg. Med. Chem..

[97] Samal S.K., Routray S., Veeramachaneni G.K., Dash R., Botlagunta M. (2015). Ketorolac salt is a newly discovered DDX3 inhibitor to treat oral cancer. Sci. Rep..

